# Patients’ mHealth Apps Usage and Data Privacy, Security, and Confidentiality Concerns: Exploratory Study

**DOI:** 10.2196/83363

**Published:** 2026-05-20

**Authors:** Nasser Alhammad, Mohannad Alajlani, Alaa Abd-alrazaq, Theodoros N Arvanitis, Gregory Epiphaniou

**Affiliations:** 1 Department of Health Informatics, Saudi Electronic University, Jeddah, Saudi Arabia; 2 Institute of Digital Healthcare, Warwick Manufacturing Group (WMG), University of Warwick, Millburn House, Coventry, CV4 7AL, United Kingdom,; 3 AI Center for Precision Health, Weill Cornell Medicine, Doha, Qatar; 4 University of Birmingham, Birmingham, United Kingdom

**Keywords:** data privacy, confidentiality, security, health care, patients, mobile health, mHealth

## Abstract

**Background:**

The Technology Adoption Model (TAM) offers a potential framework for elucidating the relationships between data privacy or security concerns and behavioral intention, perceived usefulness (PU), and perceived ease of use (PEOU) of mobile health (mHealth) apps, particularly for patients’ self-care management. In Saudi Arabia, limited information is available on these pertinent research areas despite the government’s relentless efforts to bolster the use of mHealth apps.

**Objective:**

This study applies the TAM and the psychosociocultural framework to explore the influence of patients’ data privacy and security concerns on the PU, PEOU, and behavioral intention to use mHealth apps for self-care management in Saudi Arabia.

**Methods:**

A cross-sectional study was conducted by recruiting patients using mHealth apps for self-care from various provinces in Saudi Arabia. Research instruments were developed based on the components of 2 theories: the psychosociocultural framework and TAM, which were then piloted, validated, and distributed to participants via Google Forms. Linear regression models were performed to test the hypothesized relationships.

**Results:**

Overall, 567 patients using mHealth apps participated in the study. Slightly more than one-third (217/567, 38.2%; range 35.6%‐41.4%) of the participants expressed a high level of concern regarding data privacy, confidentiality, and security, with significant predictors being female gender, higher educational qualifications, and younger age groups (<46 years). About 18% to 25% of the variance in PU, PEOU, and behavioral intention to use mHealth apps was explained by the tested factors. Patients were more likely to have higher PU following a unit decrease in data confidentiality (*β*=.31; *P*=.01) and security concerns (*β*=.47; *P*=.01). The PEOU of mHealth apps increased as users demonstrated less concern regarding data privacy (*β*=.18; *P*=.001), confidentiality (*β*=.24; *P*<.001), and security (*β*=.43; *P*=.02). Likewise, behavioral intention to use mHealth apps also increased significantly following a reduction in respondents’ concerns toward data privacy (*β*=.18; *P*=.02), confidentiality (*β*=.24; *P*=.03), and security issues (*β*=.36; *P*=.01).

**Conclusions:**

Specific demographic factors and concerns regarding data security and privacy influence patients’ PU, PEOU, and behavioral intention to use mHealth apps for self-care management. Targeting the age-, education-, and gender-based differences regarding the usage of mHealth apps. Health care providers and policymakers may consider age-, education-, and gender-based differences when developing strategies to improve the adoption of mHealth apps among the Saudi patient population.

## Introduction

### Background

The usage of mobile health (mHealth) apps has witnessed exponential growth among the general public and health care providers, especially with the introduction of smartphones [1] and the growing interest in the health care industry and research field [2]. COVID-19 is also a strong factor contributing to the heightened reliance on digital health [3]. The application of mHealth apps ranges from disease management to self-care, which constitutes activities performed to prevent or manage health conditions and promote good health. These activities include information gathering, supervising behavioral changes, managing fitness, and reminding patients of rehabilitation schedules and medication [4]. mHealth apps also assist in managing health records and providing easy access and avenues to perform mobile or remote consultations [5].

Providing timely consultation and decision-making at the point of care requires various resources, including clinical data. As a result, there is continuous advocacy for mHealth apps to be adopted by health care professionals and patients [6]. With advancements in digital health care and technologies, widespread usage of mHealth apps for self-care is expected in the next decade [7]. However, issues related to data privacy, security, and confidentiality are well-established barriers to the adoption of mHealth apps in health care settings [6-8].

Data security refers to legislative, physical, or mechanical tools used to prevent personal health information from unauthorized disclosure [8], whereas data confidentiality refers to the responsibility of those collecting data or information (ie, app developers and providers) in upholding the concerns of patients or users to whom such information is related [8]. The National Committee on Vital and Health Statistics described privacy as an individual legal right or freedom to protect or disclose their health information. Data protection in mHealth apps is crucial given the susceptibility of keyed-in information and the growing interest of attackers and hackers in mobile apps [7]. Furthermore, data breaches have been associated with a variety of factors, such as data privacy disclosure, data management and storage, data encryption, data integration, app operability, and authentication [7-9]. Patients possess a contractual relationship with health care providers, whereby the latter are responsible for ensuring the confidentiality and safety of the patient’s health information [4]. Accumulated evidence suggests low awareness among mHealth app users regarding data privacy, security, and confidentiality. Meanwhile, health care providers are more conversant with these issues, particularly as gleaned from studies conducted in Western countries [4,10].

In the Middle East, Saudi Arabia has one of the fastest-growing health care economies, which is linked to the digitization of the sector [11]. Events such as the high penetration of smartphones, the internet, and the widespread usage of social networking in the Arabian Gulf contribute to advancements in health care delivery. Aligning with the increasing number of smartphone users, numerous mHealth apps were introduced in Saudi Arabia to meet the country’s Vision 2030 goals [12,13]. The Ministry of Health has also developed specific mobile apps to facilitate self-care management and consultations between health care providers and patients [12,14].

Despite the diverse efforts put in place by the government, the uptake of mHealth apps in Saudi Arabia has yet to achieve the projected benchmark [13]. Recent studies highlighted privacy and security issues regarding data shared via mHealth apps, with the potential to cause data breaches and severe economic consequences [11,15]. However, it remains unknown whether such concerns influence the adoption and usage of mHealth apps among patients, especially for self-care management. Addressing this knowledge gap is pertinent to addressing users’ needs and developing patient-friendly mHealth apps tailored for self-care among patients in Saudi Arabia. The present empirical study explores the influence of data privacy and security concerns on patients’ perceived usefulness (PU), perceived ease of use (PEOU), and behavioral intention to use mHealth apps for self-care management.

### Research Model and Hypothesis Development

Data privacy and security concerns are among the most widely used variables in research related to mHealth systems and the adoption of various related apps. As information technology continues to increase its capacity for storing, processing, and exploring personal health information and data, researchers have developed an interest in capturing shortfalls related to privacy and data breaches [16]. Wilkowska and Ziefle [17] explored the perceived relevance of privacy and security aspects among different groups, followed by assessing the predictive power of the variables on the acceptance of medical assistive technologies. Multivariate regression models revealed that the most predictive security and privacy domains were crucial for the acceptance and usage of the technologies. Nevertheless, there is a paucity of data regarding users’ privacy concerns and their effects on mHealth app adoption, especially in the Saudi context.

The literature posits that users’ awareness of data privacy makes them circumspect about adopting technology and sharing their personal information [4,6]. Users’ perspectives and concerns about data privacy of health information may influence their avoidance of using specific health care services, including mHealth in the present context [9,18-20]. Previous research revealed that the failure to mitigate customers’ privacy concerns severely impacts customers’ behavior and attitudes toward health care services [21-24]. Mukherjee et al [23] also found that security related to privacy, combined with shared values, was positively associated with customers’ behavioral intention. The present study aligns with the argument that privacy, security, and confidentiality concerns are related to end users’ assessment of a lack of reliance on mHealth apps, especially relating to sharing their personal data.

Empirical studies have demonstrated how Technology Adoption Model (TAM) components influence the perception of using mHealth apps [25,26]. Despite the usefulness of mHealth apps for patients, Mangkunegara et al [25] found that patients were more likely to have a negative perception when such apps are difficult to operate, thereby reducing their intention to use them. Verissimo et al [26] revealed that users prioritized mHealth apps that can be easily used to retrieve health information of interest. Gagnon et al [27] posited that PEOU has a positive relationship with the adoption of health care technologies.

In terms of the acceptance of mHealth apps among the Saudi population, this study used TAM to derive the research hypotheses. The 2 key components of TAM, PEOU and PU, have been shown to influence users’ behavioral intentions [24]. However, despite extensive research on data privacy and security in the health care context, their effects on privacy concerns remain underreported [25]. A study found that higher privacy and security concerns played a negative mediating role in the association between users’ perceived risk and attitude [28]. It is plausible to predict that patients will not find usefulness in technology with a high risk of invading their privacy. Thus, privacy and security concerns might decrease individuals’ PEOU and PU for any service, including mHealth apps.

**Figure 1.**
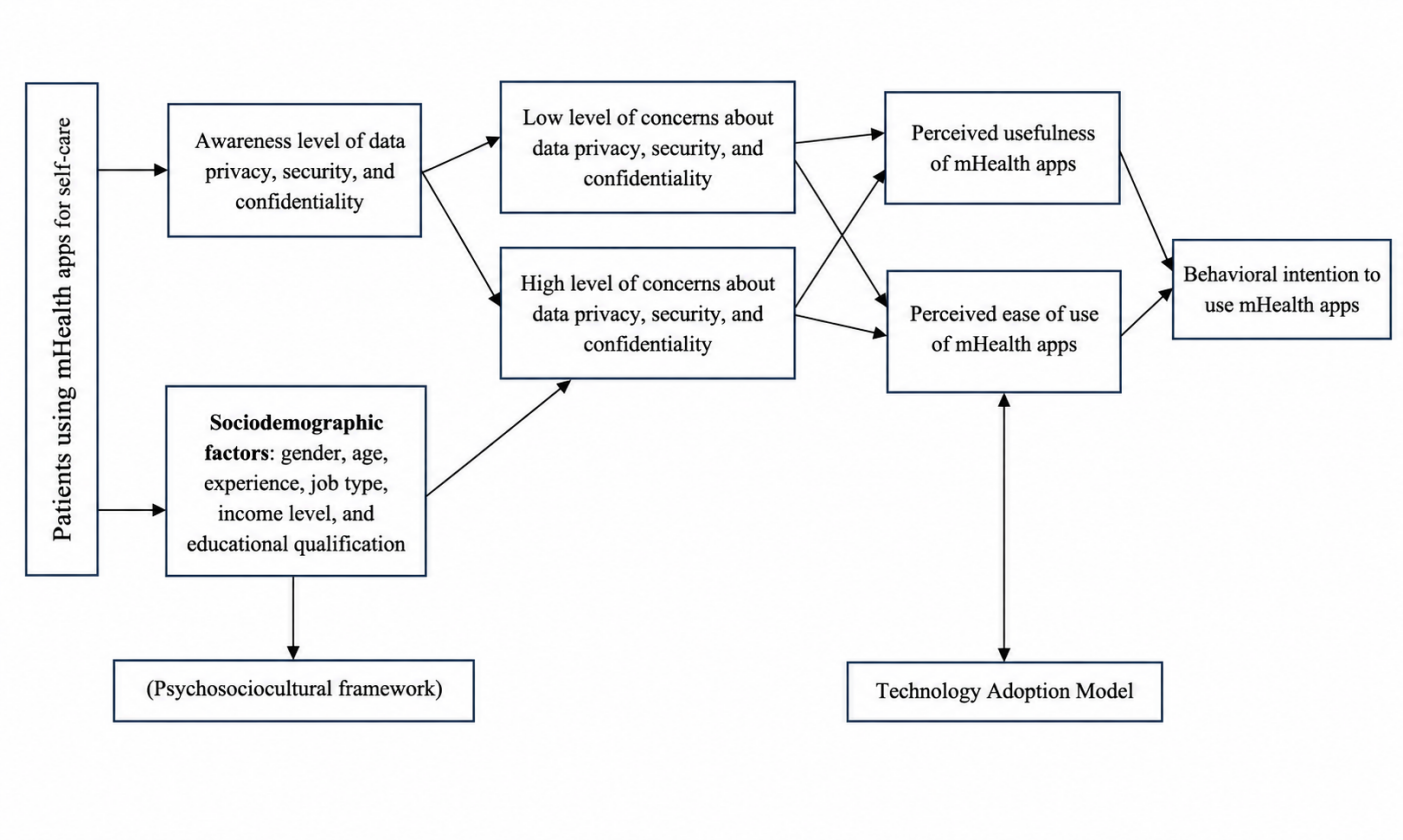
Conceptual framework showing the proposed relationship between sociodemographic factors, Technology Adoption Model dimensions and concerns regarding data privacy, security, and confidentiality. mHealth: mobile health.

Privacy and security concerns are also linked to PU because users may be unsure that health-related outcomes will be achieved if the fear of unauthorized access to their personal information cannot be guaranteed [29]. Likewise, privacy and security concerns are related to PEOU, given that addressing these issues reduces the effort that would otherwise be required in monitoring the system. Thus, security and privacy concerns constitute perceived risk, an important variable for mHealth use in terms of distance and interaction between patients and health care providers. In the mHealth context, users face the possibility of suffering a loss while using the technology [30], with most perceiving risk when there is no avenue to verify the security of the infrastructure for securing their personal health information. Patients’ perception of risk, which entails privacy and security concerns, is negatively linked to their intention to transact. Thus, privacy and security concerns might limit PEOU and negatively impact individuals’ PU for any service. It is important to elucidate these events in the context of using mHealth apps for self-care management, as detailed data and information are required to operate the digital technology. Based on these arguments, the following hypotheses are proposed:

- H1: Patients’ concerns about data privacy influence their PU, PEOU, and behavioral intention to use mHealth apps.
- H2: Patients’ concerns about the security of mHealth apps influence their PU, PEOU, and behavioral intention to use mHealth apps.
- H3: Patients’ concerns about the confidentiality of data shared on mHealth apps influence their PU, PEOU, and behavioral intention to use mHealth apps. The proposed research model is presented in Figure 1.

## Methods

### Study Area, Study Design, and Study Population

There are a total of 13 provinces in Saudi Arabia, namely Riyadh, Madinah, Mecca, Tabuk, Najran, Hail, Northern, Eastern, Al Jouf, Asir, Qassim, Jazan, and Al Baha. The study design entailed a cross-sectional method by gathering information from patients using mHealth apps for self-care management during a specific period. The study population and unit of analysis in this study encompass all patients who fulfill the inclusion criteria, particularly those who are actively using mHealth apps to participate in self-care activities for disease management.

### Inclusion and Exclusion Criteria

Certain inclusion criteria were considered to recruit the appropriate respondents. The target participants included patients aged 18 years and older, with a confirmatory diagnosis of a health condition that requires monitoring by health care providers. Participants must also be conversant with mHealth apps specifically designed for self-care management. To ensure that patients provide factual responses and minimize recall bias, they must be either current and active users of mHealth apps or have used them within 1 year prior to the time of this study. Consistent data synthesis was facilitated by defining self-care as “activities performed on mHealth apps to prevent or manage health conditions and promote good health, which encompass information gathering, supervising behavioral changes, managing fitness, setting reminders for rehabilitation schedules and medication, as well as direct consultation with clinicians or health care providers [4].”

### Instrument Development and Administration

A multidimensional approach was applied to develop the survey, which entailed an in-depth literature review, modification of existing research instruments, and discussion among the researchers. We developed a structured questionnaire, broadly divided into 2 parts. The first page in part 1 comprised the consent form and participant eligibility criteria. Detailed information on the consent form is provided in the next section. As for inclusion criteria, potential respondents were instructed to state whether they are actively using mHealth apps for self-care or had used such technologies in the last year, and the specific self-care activity performed. The remaining part of part 1 entails demographic data such as age, gender, marital status, occupation, income level, education level, current health app usage, and frequency of mobile app use.

Part 2 of the questionnaire consists of 7 sections. The first section comprised 5 items, focusing on patients’ awareness of data privacy, security, and confidentiality [15]. The second (8 items), third (6 items), and fourth (7 items) sections emphasize patients’ privacy, confidentiality, and security concerns, respectively. These items were adopted from studies conducted by Aljedaani et al [15], Zhou et al [31], and Zhou et al [4]. The PU of mHealth apps was explored in the fifth section (4 items), whereas PEOU was evaluated in the sixth section. Three items were designed in the seventh section to assess patients’ behavioral intentions. All sections and items were measured using a 5-point Likert scale ranging from 1=strongly disagree, 2=disagree, 3=neutral, 4=agree, to 5=strongly agree.

All questions were closed-ended. The questionnaire was translated from English to Arabic by 2 experienced translators. Subsequently, both versions of the questionnaire were piloted and validated. Expert opinions and pilot testing were used to prevent potential ceiling and floor effects from the questionnaire items that could affect accurate data interpretation. After validating the instrument and making adjustments based on experts’ suggestions and recommendations, the reliability of the instrument was assessed. A total of 20 patients were recruited for the pilot study, as suggested by Lackey and Wingate [32]. These respondents were not included in the final survey. Reliability was evaluated by computing Cronbach α, which indicates participants’ comprehension of each item in the questionnaire. Table 1 presents the results of the reliability analysis for each section and the overall survey instrument.

**Table 1.** Results of the reliability analysis.

Section	Number of items	Cronbach α
Awareness of data privacy, security, and confidentiality	5	0.80
Data privacy	8	0.82
Data confidentiality	6	0.86
Data security	7	0.74
Perceived usefulness	4	0.77
Perceived ease of use	4	0.69
Behavioral intention	3	0.68
Overall	37	0.81

All sections of the questionnaire fulfilled the minimum required value (Cronbach α=0.60) for acceptable internal consistency and reliability. The construct on behavioral intention yielded the lowest value (Cronbach α=0.68), while the construct on data confidentiality had the highest reliability coefficient (Cronbach α=0.86). The instrument achieved an excellent level of reliability, with an overall Cronbach α of 0.81. Hence, no item was deleted or added to any of the constructs, and the instrument was considered suitable for the final survey. The developed questionnaire (Multimedia Appendix 1) was then prepared using Google Forms and Qualtrics. Subsequently, the link was distributed electronically on different media, such as email and social networks, including WhatsApp, Facebook, and LinkedIn (Microsoft).

### Ethical Considerations

Ethical approval for this research was obtained from the Biomedical and Scientific Research Ethics Committee, University of Warwick (BSREC 03/22‐23), the Medical Research and Ethics Committee, and the Ministry of Health in Saudi Arabia. Participants were instructed to provide written and signed consent on the first page of the instrument before responding to the questions. Participation was anonymous to ensure that participant confidentiality was maintained. No identifying details were recorded, and participants were informed that they could withdraw from the study at any time without any penalty. All collected data were stored on a personal computer with a secure password and were accessible only to authorized parties (the supervisory team and the researcher). Participants did not receive any financial compensation or incentives for participation in this study.

### Recruitment

The survey was distributed in all 13 provinces in Saudi Arabia to eligible participants identified via contact repositories available at 2 hospitals purposively selected in each province. Likewise, participant sampling was nonprobabilistic and convenient. A total of 567 valid questionnaires were collected from the online survey between January 2024 and June 2024.

### Data Analysis

Descriptive statistics were used to summarize the participants’ background information. All research variables were checked for normality using the Kolmogorov-Smirnov test and presented as mean (SD) or median (IQR), as appropriate. Bivariate analysis was conducted using either Pearson chi-square test or independent *t* test to determine the association between respondents’ demographic factors and their scores for concerns regarding data privacy, security, and confidentiality. Meanwhile, linear regression analyses were conducted in 2 stages, simple and multiple, to investigate the influence of respondents’ concerns about data privacy, security, and confidentiality on PEOU, PU, and behavioral intention toward mHealth apps. For the former stage, 1 independent variable was introduced into the regression model at a time, and the relationship was assessed for its significance based on a *P* value of .10. This *P* value was chosen as a cutoff to prevent prematurely discarding key variables in the model, as well as preventing type II errors and identifying potential confounders. Thereafter, all significant independent variables were introduced into the multiple regression model. This model was used to determine how well the set of independent variables (concerns about data privacy, security, and confidentiality) predicts the dependent variables (PEOU, PU, and behavioral intention toward mHealth apps). In addition, regression analysis was used to identify the best predictor among the significant independent variables. For each model, the *R*^2^ value describes how much of the variance in the outcome variable is explained by the model, whereas standardized regression coefficient values reflect the contribution of each variable. A significant relationship was set at a *P* value of .05. All the analyses were conducted in SPSS version 25 statistical software (IBM).

## Results

### Participants’ Demographic Profile

A total of 567 valid and completed questionnaires were retrieved and analyzed. As shown in Table 2, the majority of participants were male patients (379/567, 66.8%), had a bachelor’s degree (262/567, 46.2%), and were aged 26 to 35 years (211/567, 37.2%). Slightly more than half of the participants belonged to the middle socioeconomic class (299/567, 52.5%), earning between 5000 and 14,999 Saudi Arabia riyal (SAR), compared with high-income (113/567, 20%) and low-income levels (155/567, 27.5%), respectively. Most patients had been using mHealth apps for 1 to 3 years (213/567, 38%), and approximately 94% (533/567) were active users of mHealth apps, compared with 6% (34/567) who had stopped using the digital health technology less than 1 year ago. In terms of self-care activities, most patients used the apps for monitoring behavioral or lifestyle changes (272/567, 48%), while 22% (125/567) and 28% (159/567) engaged mainly in remote consultation with health care providers and physical activities or exercise, respectively.

**Table 2.** Demographic characteristics of the participants.^a^

Variables	Value, n (%)	
Gender		
Male	379 (66.8)	
Female	179 (31.6)	
Prefer not to say	9 (1.6)	
Educational qualification		
High school or secondary	71 (12.5)	
Bachelor’s degree	262 (46.2)	
Masters’s degree	125 (22)	
Diploma	70 (12.3)	
PhD	39 (6.9)	
Age (y)		
18‐25	65 (11.5)	
26‐35	211 (37.2)	
36‐45	183 (32.3)	
46‐65	103 (18.2)	
Above 65	5 (0.9)	
Income level or socioeconomic status		
High (>15,000 SAR^b^)	113 (20)	
Middle (>5000‐14,999 SAR)	299 (52.5)	
Low (<5000 SAR)	155 (27.5)	
Experience using mHealth apps		
<6 months	113 (20)	
6‐12 months	102 (18)	
1‐3 years	213 (37.5)	
>3 years	139 (24.5)	
mHealth user category		
Active	533 (94)	
Inactive	34 (6)	
Self-care activities		
Health-related reminders	85 (15)	
Monitoring behavioral or lifestyle changes	272 (48)	
Physical activities or exercise	159 (28)	
Remote consultation	125 (22)	

a Self-care activities were assessed using multiple-choice options; therefore, the cumulative percentage is greater than 100%.

b SAR: Saudi Arabia riyal (1 SAR=US $0.27).

### Patients’ Perspectives on Data Privacy, Security, and Confidentiality

Descriptive results of participants’ responses to items used in assessing their data privacy, security, and confidentiality concerns are summarized in the Multimedia Appendix 2. Slightly more than half of the participants were concerned when health care providers asked them for too much personal information, suggesting that health care providers may be collecting excess personal information. A high proportion (394/567, 69.5%) agreed with the possibility of unauthorized individuals accessing their personal information. Most patients prioritized their data privacy when using mHealth apps (349/567, 61.6%), always checked and read the privacy policy before signing up for mHealth apps (280/567, 49.4%), and checked the privacy settings before use (277/567, 48.9%). Furthermore, although 40% (226/567) of participants agreed that app developers ensure data confidentiality, 35% (198/567) disagreed with the statements. Nevertheless, most participants reported that they always log out of mHealth apps after use, refuse to allow their data to be used for marketing research purposes, and are indifferent to sharing their clinical history on mHealth apps. Most patients (309/567, 54.6%) also agreed that a law exists to protect the usage of their personal data by a third party without their permission.

For data security, more than 60% (340/567) of the participants agreed that their mobile phones are protected with a password and/or biometrics, avoid using their smartphones with other users, and prefer mHealth apps to have user authentication to secure their personal data. In contrast, comparatively lower proportions agreed with changing their passwords regularly to avoid data hacking (246/567, 43.4%), using mHealth apps with an option to terminate permission of data collection before signing up (281/567, 49.6%), and being aware of encryption functions to assist in securing their data (234/567, 41.3%).

The mean (SD) score for data security was 3.22 (1.12), for data confidentiality was 3.04 (1.25), and for data privacy was 2.93 (1.08). Despite 38.2% (217/567) of patients demonstrating greater concerns regarding at least one of the investigated issues, no significant difference was observed in the proportions of patients expressing low or high concerns (Table 3).

**Table 3.** Data privacy, security, and confidentiality concerns and mHealth app usage.^c^

Variables	Mean (SD)	Concerns	
		Low (%)	High (%)
Data privacy	2.93^b^ (1.08)	58.6^b^	41.4
Data confidentiality	3.04^b^ (1.15)	60.8^b^	39.2
Data security	3.22^b^ (1.25)	64.4^b^	35.6
Overall	3.06 (1.12)	61.2	38.2

a Higher mean scores reflect less concern regarding data privacy, confidentiality, and security. High and low categories were computed based on a cutoff point of 2.5 on the 5-point Likert scale.

b Mean values with the same superscript letter are not statistically significantly different (*P*>.05).

A descriptive analysis of the TAM dimensions is shown in Table 4. PU recorded the highest mean (SD) score of 3.21 (1.33), followed by PEOU at 3.32 (1.33) and behavioral intention at 3.21 (1.33). These results indicate that most patients had a positive perception of the 3 domains, as evidenced by the frequency distribution, where more than 60% (340/567) were in the “high” category. No significant difference was observed in the mean scores between any of the TAM dimensions.

**Table 4.** Descriptive analysis of Technology Adoption Model dimensions^a^.

Variables	Mean (SD)	Categories	
		High (%)	Low (%)
Perceived usefulness	3.40^b^ (1.35)	68^b^	32
Perceived ease of use	3.32^b^ (1.33)	66.4^b^	33.6
Behavioral intention	3.21^b^ (1.33)	64.2^b^	35.8

a Overall, *P* values were not computed for these variables, as they measure distinct dimensions of technology adoption. Higher mean scores reflect greater perceived usefulness, ease of use, and behavioral intention to use mHealth apps. High and low categories were computed based on a cutoff point of 2.5 on the 5-point Likert scale.

b Mean values with the same superscript letter are not statistically significantly different (*P*>.05).

### Influence of Patients’ Demographic Profile and Data Privacy, Security, and Confidentiality Concerns

Table 5 depicts the bivariate analysis of patients’ demographic factors and responses to items regarding mHealth app privacy, confidentiality, and security. Female users demonstrated significantly higher mean scores (*P*<.05) in terms of data privacy, security, and confidentiality compared with male users of mHealth apps. Likewise, patients with secondary or high school qualifications and older age groups (46‐65 y and above 65 y old) recorded significantly higher scores (*P*<.05) for all investigated outcomes relative to those with higher educational qualifications (bachelor’s, master’s, and PhD) and younger patients (18-25 y old), respectively. In contrast, no significant relationships were observed in terms of income level, user category (active vs inactive), experience using mHealth apps, or type of self-care activities.

**Table 5.** Bivariate analyses of patients’ demographic profile and security, privacy, and confidentiality concerns of data shared via mHealth apps.

Variables	Data privacy		Data security		Data confidentiality	
	Mean (SD)	*P* value	Mean (SD)	*P* value	Mean (SD)	*P* value
Gender		<.001		<.001		.003
Male	2.81 (1.16)		3.08 (1.35)		2.88 (1.23)	
Female	3.20 (0.80)		3.53 (0.93)		3.36 (0.88)	
Educational qualification		<.001		<.001		<.001
High school or secondary	3.42 (1.31)		3.50 (1.44)		3.51 (1.48)	
Bachelor’s degree	3.05 (1.03)		3.23 (1.10)		3.20 (1.18)	
Master’s degree	3.04 (0.86)		3.10 (0.95)		3.11 (1.05)	
Diploma	2.89 (1.25)		3.03 (1.25)		3.01 (1.42)	
PhD	2.94 (1.02)		2.92 (1.17)		2.99 (1.22)	
Age (y)		<.001		<.001		<.001
18‐25	2.28 (1.36)		2.37 (1.43)		2.39 (1.47)	
26‐35	2.99 (1.00)		3.07 (1.11)		3.29 (1.17)	
36‐45	2.97 (1.02)		3.06 (1.10)		3.27 (1.21)	
46‐65	3.14 (0.98)		3.32 (1.01)		3.49 (1.11)	
>65	3.12 (0.82)		3.60 (0.48)		4.30 (0.57)	
Income level		.24		.38		.35
High	3.20 (0.80)		3.55 (0.87)		3.22 (0.78)	
Middle	3.12 (1.31)		3.23 (1.25)		3.50 (1.57)	
Low	3.02 (1.03)		3.11 (1.10)		3.42 (1.24)	
Experience in using mHealth^a^ apps		.43		.72		.50
<6 months	2.89 (1.25)		3.03 (1.25)		3.22 (1.42)	
6‐12 months	2.94 (1.02)		3.08 (1.17)		3.20 (1.22)	
1‐3 years	2.94 (1.09)		3.23 (1.24)		3.04 (1.16)	
>3 years	2.92 (1.06)		3.22 (1.26)		3.03 (1.15)	
mHealth app user category		.87		.42		.50
Active	2.89 (1.00)		3.01 (1.02)		3.49 (1.71)	
Inactive	2.92 (1.02)		3.11 (1.30)		3.32 (1.26)	
Self-care activities		.62		.26		.32
Health-related reminders	2.72 (1.04)		3.04 (1.00)		3.10 (1.18)	
Monitoring behavioral changes	2.84 (1.01)		2.99 (1.14)		2.95 (1.26)	
Physical activities	2.75 (0.88)		2.94 (1.12)		3.00 (1.01)	
Remote consultation	2.68 (0.78)		2.82 (0.58)		3.22 (0.77)	

a mHealth: mobile health.

### Association Between TAM Dimensions and Patients’ Privacy, Security, and Confidentiality Concerns

Table 6 presents the 3 final regression models for the relationship between TAM dimensions and data privacy, security, and confidentiality concerns. As shown in model 1 (PU), approximately 26% of the variance in PU of mHealth apps was explained by the tested factors. There was no significant linear relationship between data privacy concerns and the PU of mHealth apps (*P*=.18). However, there was a significant positive relationship between the PU of mHealth apps and concerns regarding data confidentiality (*P*<.001) and security (*P*<.001). Thus, patients were more likely to have higher PU following a unit decrease in data confidentiality and security concerns.

**Table 6.** Multiple regression models showing the influence of perceived usefulness, perceived ease of use and behavioral intention to use mobile health apps and concerns regarding data privacy, confidentiality, and security.

Variables	*β*	*t* test (*df*)	*P* value	95% CI
Model 1 (PU^b^)^c^				
Constant	—^d^	−0.28 (563)	.78	−0.18 to 0.14
Data privacy	.06	1.33 (563)	.18	−0.04 to 0.12
Data confidentiality	.31	7.42 (563)	.01	0.27 to 0.46
Data security	.47	10.82 (563)	.01	0.42 to 0.61
Model 2 (PEOU^e^)^f^				
Constant	—	−0.18 (563)	.85	−0.17 to 0.14
Data privacy	.188	3.98 (563)	.001	0.11 to 0.34
Data confidentiality	.248	5.75 (563)	.001	0.18 to 0.38
Data security	.436	9.76 (563)	.02	0.37 to 0.55
Model 3 (Behavioral intention)^g^				
Constant	—	−0.21 (563)	.82	−0.20 to 0.160
Data privacy	.18	3.25 (563)	.02	0.08 to 0.35
Data confidentiality	.24	4.80 (563)	.03	0.16 to 0.39
Data security	.36	7.02 (563)	.01	0.282 to 0.50

a Degrees of freedom for the reported *t* values were based on the residual degrees of freedom from the regression models.

b PU: perceived usefulness.

c Model 1: *R*2=0.26; adjusted *R*2=0.25; SE=0.12.

d Not applicable.

e PEOU: perceived ease of use.

f Model 2: *R*2=0.20; adjusted *R*2=0.19; SE=0.09.

g Model 3: *R*2=0.22; adjusted *R*2=0.21; SE=0.11.

In terms of PEOU of mHealth apps, about 20% of the variation in PEOU of mHealth apps was explained by the independent variables. Specifically, the PEOU of mHealth apps increased as users demonstrated less concern regarding data privacy, confidentiality, and security (ie, higher mean scores for each independent variable). All the tested factors demonstrated significant linear relationships (*P*<.05) with behavioral intention to use mHealth apps. Behavioral intention to use mHealth apps increased significantly following a reduction in respondents’ concerns toward data privacy, confidentiality, and security issues. The *R*^2^ value revealed that 22% of the variance in behavioral intention to use mHealth apps was explained by the tested factors.

## Discussion

### Main Findings

Given the widespread use of smartphones and the adoption of digitalized health technologies in Saudi Arabia, it is pertinent to elucidate events that may shape the adoption and usage of mHealth apps, particularly those designed for self-care management. Examples include several mHealth apps implemented by the Ministry of Health in response to COVID-19 [33,34] and those for managing chronic diseases, such as the Cora Health and Sehhaty Wa Daghty apps [35,36]. The provision of adequate security and privacy is key to enhancing the effectiveness and adoption of these apps [35]. However, relevant privacy and security issues related to mHealth are poorly understood in Saudi Arabia [11]. This study provides insight into the factors influencing mHealth app usage for self-care among patients and their data privacy, security, and confidentiality concerns.

Approximately one-third of the patients (217/567, 38.2%) conveyed greater concerns regarding the privacy and security of mHealth apps used for self-care in Saudi Arabia. This finding supports the reports from prior local studies, whereby users perceived the privacy of mHealth apps as inadequate, with a greater risk of affecting data security [11,35]. Further comparison can be made between our study and that of Aljedaani et al [15], who focused on end users’ awareness of security features in mHealth apps introduced in Saudi Arabia. Most respondents in their study were unaware of the existing security features, which led to only a few respondents expressing concerns regarding data privacy. The discrepancy stems from the fact that patients using mHealth apps for self-care were enrolled in our study and are well conversant with the technology. Our results are also similar to research conducted in Western countries. For instance, 96% of users in the United Kingdom expressed concern about personal data security and privacy, leading to requests for user authentication and data encryption [37].

Gender-, education-, and age-based differences were observed in relation to concerns regarding mHealth apps’ data privacy and security. Notably, female patients in our study were more concerned about data privacy and security compared with male patients. While the underlying reasons for these results are not well understood, given the lack of published research on the topic, a few studies have shown gender-specific differences in satisfaction levels with mHealth apps [36]. For instance, male users reflect higher satisfaction levels with mHealth apps and perceptions of health-related technologies [38-40]. In our study, female patients were less satisfied with mHealth apps, which may account for their concerns regarding the security and confidentiality of data shared on digital technologies.

Younger patients also reflected a higher level of concern about privacy in mHealth apps relative to the older age groups. This result might be influenced by increased exposure to events relating to data breaches, as they spend more time on their smartphones compared with the older population [39,40]. Moreover, young patients tend to be more familiar with digital interfaces, as evidenced in research reporting mHealth app usage for diabetes self-management [41] and studies conducted in Canada [42] and the United Kingdom [43]. As a result, young patients have greater inclinations toward security notifications and authentication, increasing their perception of data privacy and security [36]. Higher technological acceptance and comfort among young users, variation in user expectations and technological literacy levels, and their effects on overall satisfaction may explain these findings.

Technology literacy levels have been demonstrated as a predictor of attitude and intention to use mobile apps for self-care management, such as diabetes and hypertension control [42], but it is unclear whether educational status shapes patients’ perspectives on data privacy. In this study, patients with higher educational qualifications were more likely to raise concerns regarding data privacy and security compared with less educated patients. The reason for this finding may stem from greater confidence and more informed decision-making when using mHealth technologies among highly educated users. Higher education could also influence patients’ attitudes or intentions to use mHealth apps for self-care [44]. A recent study in Saudi Arabia found no significant association between diabetes patients’ educational status and their attitude or intention to use mHealth apps [45,46]. Our study sheds more light on the topic, highlighting how educational level and privacy concerns may interact in shaping patients’ intentions to use digital health technologies, particularly in the Saudi Arabian context. Overall, the demographic influences identified in the present study underscore the significance of tailoring mHealth apps that take into account diverse patient preferences and abilities to maximize effectiveness and engagement.

As for the TAM domains, the hypothesized relationship between PU of mHealth apps and patients’ concerns about data security and confidentiality was supported. Greater concerns about data security, privacy, and confidentiality influenced patients’ PEOU of mHealth apps. Hence, patients found mHealth apps to be more useful and easy to operate upon perceiving the technology as highly secure and confidential in handling their personal data. In other words, 1 approach to address users’ concerns regarding data security is to ensure they can easily navigate the technology and access the security features. Thus, apart from improving the security features as suggested by most users, ensuring that these security features are easy to use is even more important. These findings are consistent with studies reporting data security issues as reasons for users declining to adopt digital health technologies [36,46]. Prior studies depicted that security and privacy are fundamental aspects when designing self-care mobile apps for users in Saudi Arabia [15,36]. For instance, the Sehhaty Wa Daghty app, a widely used mHealth app in Saudi Arabia, has effective provisions for gathering and handling users’ personal health information by following principles outlined in the European Commission’s code of conduct on privacy of mHealth apps [36,47]. These provisions contributed significantly to a high level of adoption among the target population. Moreover, a recent empirical investigation into end users’ security awareness of mHealth apps revealed participants’ desire for usable security, such as biometric authentication, and their concerns about data anonymization [15]. Thus, the present study reflects that addressing data privacy and security issues is a promising approach to enhancing the adoption and usage of self-care mobile apps.

Lastly, patients’ behavioral intention to use mHealth apps improved upon perceiving that the technology effectively addressed data security, privacy, and confidentiality. These findings support the position of previous studies in which users’ awareness of data privacy was reported to influence their intention to adopt technology and share their personal information [4,48-50]. Behavioral modifications toward specific health care services, including mHealth, may be shaped by users’ perspectives and concerns about the data privacy of health information [9]. Past research demonstrated that severe consequences and effects on users’ behavior and attitude toward health care services may arise from the failure to mitigate customers’ privacy concerns [23]. Mukherjee et al [23] also reported that security to privacy combined with shared values were positively associated with customers’ behavioral intention, which is consistent with the present study.

### Implications of the Study

This study has important implications for mHealth app developers and the health care system in Saudi Arabia. The security and adoption of mHealth systems could be effectively strengthened if both developers’ and patients’ perspectives on mHealth apps are aligned [37]. mHealth developers can use the research findings to devise strategies to ensure the safety of patient health information, deliver health care services efficiently, and maintain a balance between security and usability. The findings from this study reflect underlying issues relating to patients’ awareness of security and privacy features provided in mHealth apps implemented by the Saudi government. Hence, the research outcomes reveal certain factors contributing to the present low adoption rate of mHealth apps among patients. Necessary adjustments in terms of decision-making, policies, and investments could be made to ensure that mHealth apps align with Vision 2030 goals, particularly for self-care management.

This study findings also have important research implications by demonstrating the relevance of combining technology adoption theories and the PSC framework in understanding factors that could shape patients’ decisions to adopt and use mHealth apps for self-care management. As only a few studies on this research topic have been published, this study offers more opportunities for exploring relevant theoretical models that could be used to explain factors contributing to patients’ views and understanding of mHealth app security and privacy.

### Limitations of the Study

Despite using a cross-sectional approach in this study, certain methodological limitations need to be acknowledged. The inclusion and exclusion criteria used in selecting the study participants might have excluded potential participants with pertinent information on the research topics. Furthermore, only patients using mHealth apps for self-care management were recruited, and the use of self-reported instruments is subject to response bias. These limitations may affect the generalizability of the results. Another possible limitation is the lack of generalizability to other digital health tools and populations of other countries, since the patients were recruited only from Saudi Arabia. Lastly, this study used a cross-sectional design, and the findings depict only the associations between the studied variables, which include patients’ demographic profile, PEOU, PU, and behavioral intention to use mHealth apps, as well as concerns regarding data privacy, security, and confidentiality. In other words, no causal relationships can be inferred. Future studies may consider including health care professionals and app developers to build on the present findings.

### Conclusion

This study revealed that about one-third of patients using mHealth apps for self-care expressed concerns regarding data privacy and security, which were shaped by demographic factors such as gender, education, and age. Available data indicated that income level, experience, user category (active vs inactive), and type of self-care activities had no influence on data privacy and security perception. Concerns regarding data security, privacy, and confidentiality shaped the PU, PEOU, and behavioral intention to use mHealth apps for self-care management. Thus, a proposed approach to address patients’ security concerns is to ensure that they can easily navigate the technology and access security and privacy features. Ensuring that these security features are user-friendly is equally important. Given the rising usage of smartphones, huge investment in mHealth apps, and the campaign to improve the usage of advanced health care systems in Saudi Arabia, this research elucidates a vital aspect that may affect these targeted goals. Policymakers and relevant bodies can use the findings to implement a model to enhance the adoption of mHealth apps, particularly by addressing patients’ concerns regarding their data privacy and security.

## Supplementary material

10.2196/83363 Multimedia Appendix 1 Questionnaire.

10.2196/83363 Multimedia Appendix 2 Information sheet and consent.

## Abbreviations

mHealth: mobile health
PEOU: perceived ease of use
PU: perceived usefulness
TAM: Technology Adoption Model
